# Symptoms and Severity of COVID-19 in Patients with Immune-Mediated Inflammatory Diseases: Experience of a University Medical Center

**DOI:** 10.1155/2024/6627035

**Published:** 2024-03-27

**Authors:** Tobias Schlosser, Marco Krasselt, Louis Elsing, Martin Hecker, Babett Holler, Albrecht Hoffmeister

**Affiliations:** ^1^Department of Oncology, Gastroenterology, Hepatology, Pneumology and Infectious Diseases, Division of Gastroenterology, University Medical Center, Leipzig, Germany; ^2^Department of Endocrinology, Nephrology and Rheumatology, Division of Rheumatology, University Medical Center, Leipzig, Germany

## Abstract

**Background:**

The pandemic situation of the novel coronavirus (severe acute respiratory syndrome coronavirus 2 (SARS-CoV-2)) and its associated disease (coronavirus disease 2019 (COVID-19)) represents a challenging condition with a plethora of aspects. The course of COVID-19 in patients with immune-mediated inflammatory diseases (IMID) such as inflammatory bowel disease (IBD) and rheumatic diseases (RD) is not well known. Our study is one step toward closing this gap by collecting data on vaccination rates, infection-free survival, and individual symptom severity.

**Methods:**

We conducted a prospective questionnaire-based study between April 2022 and October 2022 at our university hospital. Outward patients over the age of 18 years were screened for participation and reported about their infection/infection-free survival since the start of the pandemic.

**Results:**

Finally, 156 patients were included in the study, 117 (75.0%) of which had inflammatory bowel disease and 39 (25.0%) patients with rheumatic disease. Altogether, 143 (91.7%) persons had received at least one vaccination against SARS-CoV-2. A total of 153 patients provided information regarding their COVID-19 history: 81 patients (52.0%) self-reported about their SARS-CoV-2 infection. In general, courses of infection were mild: only two patients (2.5% of patients with reported COVID-19) were hospitalized due to COVID-19 with one (1.2%) of the two needing intensive care. Asymptomatic COVID-19 had been described by 7 persons (8.6% of patients with reported COVID-19). Acute COVID-19 was accompanied by fatigue/tiredness in 58 persons (71.6% of patients with history of COVID-19) as the most frequent symptom. Other complaints were common cold (55 patients = 67.9%), cough (51 patients = 63.0%), headache (44 patients = 54.3%), and fever (35 patients = 43.2%). Stratified by vaccination status (unvaccinated vs. at least once vaccinated), the time to infection differed significantly (logrank test: *p* = 0.04, Chi^2^ 4.1). At least once vaccinated people had a median COVID-19-free survival of 28.5 months (confidence interval (CI): 23.6 months-not reached). Without any vaccination, the estimated time to infection was 25.1 months (CI: 23.6 months-not reached).

**Conclusion:**

Our IMID patients have a high rate of vaccination against SARS-CoV-2. Data show a significantly longer infection-free survival in vaccinated IMID patients as compared to unvaccinated patients. Discrimination between symptoms of COVID-19 and a concomitant inflammatory disease is difficult as complaints might be overlapping. This trial is registered with DRKS00028880.

## 1. Introduction

At the end of 2019, a series of atypical pneumonia cases in Wuhan, the capital of Hubei Province in the People's Republic of China, had evolved. The cases spread, evolving to an epidemic throughout China, and finally to an increasing number of cases worldwide. Within weeks, the causal agent was found in a novel coronavirus (severe acute respiratory syndrome coronavirus 2 (SARS-CoV-2)). In February 2020, the World Health Organization named the associated infective illness coronavirus disease 2019 (COVID-19) [1].

By now (October 2023), 677 million cases of COVID-19 have been counted around the world, resulting in 6.9 million associated deaths [2]. In Germany, 37 million SARS-CoV-2 infections were reported. The German Free State of Saxony, where our study was conducted, has been heavily affected by COVID-19 with 2 million cases out of 4 million inhabitants [3].

The clinical course of COVID-19 is highly variable: a notable number of asymptomatic people (about 33%) face 14% of patients with severe disease (i.e., dyspnea and*/*or hypoxia). In 5% of cases, the disease takes a critical course with respiratory failure or shock and in 2.3% the disease ends fatally [4–6]. Not only the severity of COVID-19 but also the spectrum of symptoms varies: cough, myalgias, and headache are most frequently reported. Other commonly described symptoms are diarrhoea, sore throat, anosmia/taste disorders, and joint pain. The proven involvement of distinct organ systems has led to the understanding of COVID-19 as a multisystem disorder [7]. The critical point of pathogenesis seems to be a dysregulated immune system, which results in hyperinflammation [8, 9].

Immune-mediated inflammatory diseases (IMID) represent a broad and heterogenic group of chronic illnesses with partially overlapping pathogeneses. Even within the affected organ system, there is distinct manifestation, leading to further subclassification (e.g., gastrointestinal: Crohn's disease, ulcerative colitis; rheumatic: rheumatoid arthritis, axial spondyloarthritis, systemic lupus erythematosus, psoriatic arthritis, granulomatosis with polyangiitis; ophthalmologic: noninfectious uveitis and dermatologic: psoriasis) [10]. About 4.5% of the world's population is affected by IMID [11]. Due to the underlying condition on the one hand and immunosuppressive therapy of which, on the other hand, infectious diseases are in general more frequent and more severe in IMID patients [12, 13]. The effects of COVID-19 on this huge patient collective have been a critical aspect of the COVID-19 pandemic. Prevalence, severity, and lethality of COVID-19 seem to range in the level of the general population. Controlled IMID is regarded as being protective against severe course of COVID-19 [12, 14, 15]. Pharmacologic immunosuppression seems no risk factor for SARS-CoV-2 susceptibility [16]. A clear connection between the severity of COVID-19 and immunosuppressive agents for IMID is not established as the data are contradictory. Higher systemic steroid dosage (e.g., prednisone ≥10 mg/day) and B cell depletion by rituximab seem to be associated with worse course and outcome of COVID-19 [14, 17–23].

Due to this lack of knowledge, our center initiated this study to systemically collect missing data.

## 2. Methods

**Figure 1:**
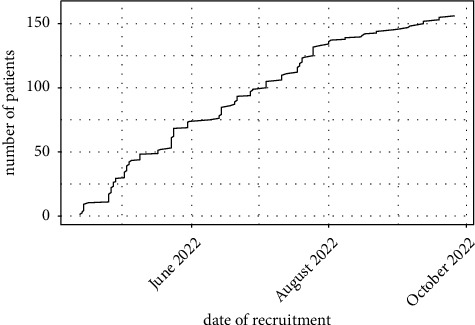
Recruitment progression.

We conducted a monocentric investigator-initiated trial at our tertiary medical center. Outpatients over the age of 18 years and established diagnosis of immune-mediated inflammatory disease (IMID) were screened. Excluded were patients not capable or willing to provide information about their SARS-CoV-2 infection/COVID-19 since the start of the pandemic. Data are collected by a self-filled form in the German language. Screening and recruitment (equals questionnaire completion) took place between April 2022 and October 2022 (Figure 1). In case a patient entered twice during a separate appointment, only the latest questionnaire got into further analysis.

The project was performed in accordance with guidelines for good clinical practice (GCP, E6/R1) and the ethical guidelines of the Helsinki Declaration. Approval was given by the local ethics committee (Faculty of Medicine, Leipzig University Medical Center, internal reference number 013/22-ek). Informed written consent was obtained from every participant. No benefit (financial/nonfinancial) was provided in return for study participation. Missing willingness or capability for participation meant no disadvantage with regard to further treatment. This study had been recorded in the German Clinical Trials Register (identity document DRKS00028880). No external funding had been raised.

Symptoms of COVID-19 were asked in multiple-choice format. A selection of common clinical features was made after literature research [24, 25]. More than one answer was possible. Additional features could be entered in written form. A special definition/threshold for a symptom (e.g., measured body temperature to be set as fever) was not given. Subjective estimation was sufficient.

Types of vaccination against SARS-CoV-2 had been checked by multiple-choice questions as well. We preselected the four authorised vaccines that had been authorised by the European Medicines Agency (EMA) at the time of study conception (December 2021): Comirnaty® by BioNTech/Pfizer, Spikevax® by Moderna Biotech, Vaxzevria® by AstraZeneca, and Jcovden® by Johnson&Johnson/Janssen-Cilag International NV [26]. A single selection for the vaccine could be made from first to fourth vaccination. Free-text fields had been retained each time.

The attending physician filled in the features regarding IMID (e.g., time of diagnosis, extent, and pharmacological therapy) on base of their knowledge and each individual's medical records. Prednisone and prednisolone doses were considered equal glucorticoid strength [27, 28].

Statistics and graphics were carried out by open access software “R,” version 4.1.1 (The R Foundation for Statistical Computing, USA). Chi-squared (Chi^2^) testing with Yates' continuity correction checked for independence between compared subgroups. A two-sided *t*-test has been performed for continuous variables. Welch modification of the *t*-test was used if Levene's test showed inequality of variance [29].

Time-to-event analysis regarding the onset of individual COVID-19 was managed by Kaplan–Meier method [30, 31]. The starting point for each individual was set to the 27th of January 2020, the date of the first proven SARS-CoV-2 infection in Germany [32]. The difference between subgroups is calculated by logrank testing [33]. The reverse Kaplan–Meier estimator calculated the median follow-up [34].

*P* value below five percent was noted as a criterion regarding statistical significance.

## 3. Results

At the end of recruitment, 170 questionnaires were used for primary analysis. After removing double entries, 156 patients were eligible for further investigation.

The majority had a diagnosis of inflammatory bowel disease (*n* = 117; 75.0%) representing 69 patients (44.2%) with Crohn's disease and 48 patients (30.7%) with ulcerative colitis. The entire cohort of immune-mediated inflammatory diseases was completed by 39 patients (25.0%) with rheumatic diseases. This heterogenic group consisted of 13 persons (8.3%) with rheumatoid arthritis, nine (5.8%) with axial spondyloarthritis, six (3.8%) with systemic lupus erythematosus, and six (3.8%) affected by psoriatic arthritis. Two patients (1.2%) had the diagnosis of granulomatosis with polyangiitis. Sjögren's syndrome, juvenile idiopathic arthritis, and dermatomyositis/polymyositis were diagnosed in one (0.6%) person each (Table 1).

**Figure 2:**
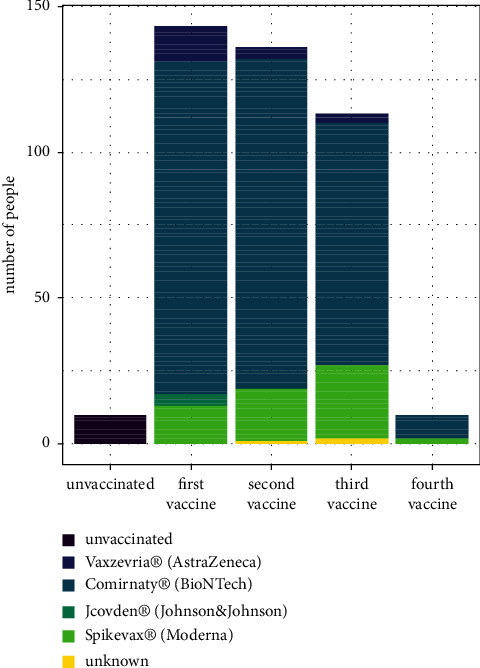
Stacked bar chart of administered vaccines against SARS-CoV-2. SARS-CoV-2: severe acute respiratory syndrome coronavirus 2.

More women (*n* = 89; 57.1%) than men (*n* = 67; 42.9%) were part of our IMID cohort. The median and mean age at the survey were 42 and 46 years, respectively. The youngest patient was aged 18 years, and the oldest patient was 95 years. Most of the patients reported at least one vaccination against SARS-CoV-2 (*n* = 143; 91.7%). Ten patients (6.4%) did not receive any vaccination against COVID-19, and three patients (1.9%) did not provide the requested information (Table 2). Administered vaccines were mainly mRNA-based with 127 persons having received Comirnaty® or Spikevax®; twelve patients had been vaccinated by a doctor with Vaxzevria®, and four patients with Jcovden® in their first course of SARS-CoV2-vaccination. A second dose of vaccination was reported by 136 IMID patients (87.1%; 4 x Vaxzevria®, 113 x Comirnaty®, 18 x Spikevax®, and 1 x Unknown). Three vaccinations were declared by 113 persons (72.4%; 3 x Vaxzevria®, 83 x Comirnaty®, 25 x Spikevax®, and 2 x Unknown). Finally, four vaccines were given to ten IMID patients (6.4%; 8 x Comirnaty® and 2 x Spikevax®; Table 3, Figure 2).

COVID-19 was self-reported in 81 (51.9%) IMID patients, and the associated SARS-CoV-2 infection was mainly detected by positive polymerase chain reaction (*n* = 68; 83.9%, Table 4). Three patients did not answer this topic, and 72 persons had no proven and/or suspected COVID-19. The severity of COVID-19 appeared rather mild with only one person in need of intensive care and one further in the inpatient setting. Among the 81 persons with a history of COVID-19, the majority (*n* = 64; 79.0%) received no special treatment and a smaller fraction (*n* = 15; 18.5%) had been treated as an outpatient (e.g., general practitioner, Table 5).

When divided into patients with a history of COVID-19 versus patients without COVID-19, it was observed that there were no significant differences in terms of gender, type of IMID (rheumatic disease vs. inflammatory bowel disease), vaccination status, and number of administered vaccinations. However, there was a difference in mean age between these subgroups, with patients who had a history of COVID-19 being younger than patients who did not report having a SARS-CoV-2 infection (42.9 years vs. 48.2 years; *p*=0.046; Table 6).

**Figure 3:**
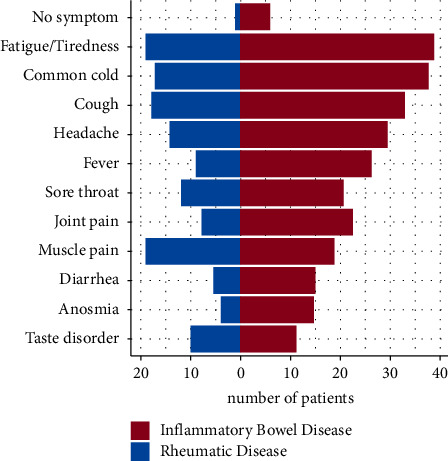
Distribution and frequency of symptoms in COVID-19. COVID-19: coronavirus disease 2019; the most frequently reported symptoms are presented, sorted decreasingly by cumulative incidence.

Predominant symptoms of COVID-19 were fatigue (*n* = 58; 71.6%) and features of common cold (*n* = 55; 67.9%). Cough (*n* = 51; 63.0%), headache (*n* = 44; 54.3%), fever (*n* = 35; 43.2%), and sore throat (*n* = 33; 40.7%) were reported commonly, as well as taste disorder (*n* = 21; 25.9%), anosmia (*n* = 19; 23.5%), vertigo (*n* = 18; 22.2%), and dyspnea (*n* = 15, 18.5%). General joint and/or muscle pain (*n* = 31/28, 38.3%/34.7%), which are also characteristic features of rheumatic diseases, were stated as a symptom during the COVID-19 course. Similar to that, diarrhoea, a typical symptom of inflammatory bowel disease, had been named as an issue in twenty COVID-19 cases (24.7%). Further symptoms were memory disorder (*n* = 13; 16.0%), dry mucosa (*n* = 12; 14.8%), chest pain (*n* = 11; 13.6%), and nausea (*n* = 10; 12.3%). Painful breathing (*n* = 4; 4.9%), hypersalivation (*n* = 3; 3.7%), and chills (*n* = 2; 2.5%) had been reported as well. One patient (1.2%) each associated chills, diffuse cognitive dysfunction, and gastrointestinal bleeding with COVID-19. Seven patients (8.6%) had no specific symptom of SARS-CoV-2 infection (Table 7, Figure 3, and Table 8 list COVID-19 symptoms with regard to rheumatic and inflammatory bowel disease).

**Figure 4:**
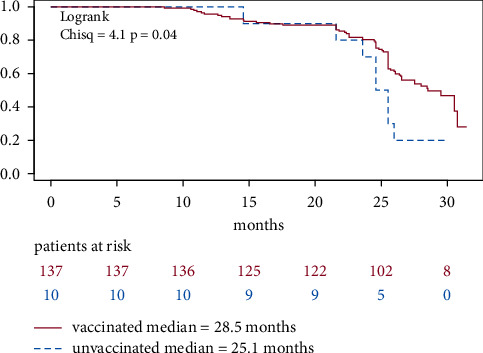
COVID-19-free survival stratified by vaccination status. COVID-19: coronavirus disease 2019; IMID: immune-mediated inflammatory disease; vaccinated = at least one vaccination against severe acute respiratory syndrome coronavirus 2.

The 81 COVID-19 cases took place between October 2020 and August 2022. We estimated a median infection-free survival of 28.5 months (CI (95% confidence interval): 26.1–not reached). Patients in the vaccinated subgroup showed a significantly longer infection-free interval compared to unvaccinated IMID patients (median: 26.1 months (CI 23.6–not reached) vs. 28.5 months (CI 26.5–not reached)) according to logrank test (*p*=0.04, 2, Figure 4, Table 9).

Pharmacological therapy for IMID was quite heterogenic in both subgroups of patients with and those without a history of COVID-19 (Table 10). Azathioprine was used in 12 patients (14.8%) with and in 11 patients (15.3%) without self-reported COVID-19 (*p*=1).

Methotrexate has been established in five patients each (6.2% with vs. 6.9 without COVID-19 history, *p*=1). In the COVID-19 cohort, three patients (3.7%) were taking hydroxychloroquine; contrary to that, one person (1.4%) was without reported SARS-CoV-2 infection. Persons on biological agents built a main group in our cohort with 35 patients (43.2%) being treated in the COVID-19 cohort vs. 36 patients (50%) with concomitant biologic and lack of COVID-19 history (*p*=0.50). An in-depth analysis of biologic agents was not computed due to the heterogeneity of pharmacological principles and relatively low absolute numbers.

Systemic glucocorticoid therapy was less frequently used within the COVID-19 cohort (13.6% vs. 30.6%, *p*=0.02) but at comparable dosages (mean 10.5 vs. 10.3 mg, *p*=0.98, Table 11).

Some therapeutic substances are only reported within one patient cohort (IBD or RD). This is due to disease-specific efficacy and/or labeled use (e.g., mycophenolate was exclusively used in RD patients).

## 4. Discussion

The ongoing COVID-19 pandemic challenges our health system on an extreme scale. A systematic report on a well-described cohort of people with immune-mediated inflammatory disease (IMID) and their individual medical history regarding COVID-19 has not yet been published. We, therefore, completed this investigator-initiated trial at our center with a remarkable number of patients to give a systematic overview of COVID-19 courses in IMID.

We report a high percentage of people with vaccination against SARS-CoV-2; 91.7% were at least once vaccinated. This finding stands out under the background of much lower immunization rates in Germany (78%) and in our federal state (66%); percentages refer to a fraction of the population, who had received at least one SARS-CoV-2 vaccine. Data were retrieved by the 26th of September 2022, representing the date of the last screened patient [35]. Given the absence of mandatory population-wide vaccination programs, we interpret this as an expression of the high will of IMID patients towards SARS-CoV-2 vaccination. The presence of an IMID as an underlying chronic disease might serve as an incentive towards vaccine administration because certain preexisting comorbidities are associated with more severe courses of COVID-19 [36, 37], and although IMID has not been shown as an independent risk factor, mainstream media reported broadly over COVID-19 severity in otherwise chronically ill patients. This might have lowered vaccination thresholds [38]. Besides, federal health recommendations addressed people with an autoimmune disorder and/or rheumatic disease within their very first nationwide publications: rheumatic and IBD patients were together considered as highly prioritised for SARS-CoV-2 vaccination [39, 40]. Furthermore, a relevant fraction of IMID patients have immunosuppressive comedication, which can be found in our cohort as well. This represents an established risk factor for severe COVID-19 and might be another individual reason towards vaccination [36, 37]. Finally, our cohort represents persons with a certain willingness for medical advice and guidance as they per se are part of our outpatient ward. This might stand for a proper health awareness and a tendency towards preventive measurements such as vaccinations. In accordance with that, high SARS-CoV-2 vaccination coverage is reported in RD patients [41].

The mRNA-based vaccines (Comirnaty® and Spikevax®) and the viral vector vaccine Jcovden® have a similar administration schedule, which includes injections of two doses within four to six weeks as a primary immunization course. The other viral vector vaccine (Vaxzevria®) only needs one administration for basic immunization, and this could be one reason that it was solely reported in the first round of vaccination (Table A8 and Figure 2). Another reason for the dominance of mRNA vaccines over vector vaccines might have been the restrictive recommendation for the latter that came up around April 2021, due to thromboembolic complications following vaccination [39]. In general, administration recommendations for all four vaccines regarding primary immunization and booster are quite similar [42]. That is why we summarized all patients, who had received at least one authorised vaccine, into one subgroup when stratified regarding self-reported COVID-19. This method, of course, neglected differences in vaccine effectiveness: a large case-control study detected a significantly higher efficacy of mRNA vaccines (Spikevax® 93%/Comirnaty® 88%) compared to the vector vaccine Jcovden® (71%) regarding COVID-19 hospitalization [43].

Our results suggest that IMID patients with at least one vaccination against SARS-CoV-2 had a longer COVID-19-free survival. High efficacy against laboratory-proven COVID-19 has been reported (Spikevax® 96%/Comirnaty® 95%/Jcovden® 75%, one month after basic immunization) [44]. Although it might be impaired by some drugs such as rituximab, vaccine efficacy was also demonstrated in patients with rheumatic inflammatory diseases under immunosuppressive treatment [45]. Interestingly, our finding seems to be supported by our analysis although several possible confounders are not considered: first of all, heterogeneity between the two groups (once vaccinated vs. unvaccinated) was not checked. Vaccination status and vaccine type have not been regularly verified by medical documentation and/or laboratory results (e.g., antibody detection). Memory bias might have impaired recall of vaccination type/status. However, we estimate that false recollection is rather unlikely in this issue, as discussions over SARS-CoV-2 vaccination dominated a broad and lively debate in the individual and society's context. Moreover, vaccination status had to be recalled on a regular basis in daily activities (access to public places). On the other hand, some people, who stated absence of COVID-19 in their history, might have been infected with SARS-CoV-2 but were not tested; e.g., due to lack of symptoms, no available test or missing will for testing because of individual reasons (to avoid quarantine). In general, social desirability might as well have influenced the answer of infection/vaccination status, as patients in this nonanonymous survey could have tended to fulfil presumed answers [46]. The time of vaccination administration was also not considered. This might explain differences in infection protection by waning immunity over time [47–50]. Additionally, different SARS-CoV-2 variants, with distinct clinical features regarding breakthrough infection and reinfection rate due to immune evasion, were predominantly circulating during a pandemic and are not recorded in our time-to-event analysis [51]. The finding that the patients with a history of SARS-CoV-2 infection had lower age than the ones without a history of COVID-19 is consistent with epidemiological data that the younger population was earlier affected within the course of the pandemic. More social interaction (educational/work duties and cultural gatherings) and fewer social restrictions for these groups could be the underlying reason [2].

The hospitalization rate seems quite low with only two out of 81 COVID-19 patients (2.5%). A higher hospitalization rate had been reported earlier with an estimation of 14% [25], but this dates back to the beginning of 2020 at a very early course of the pandemic: insecurities and concerns about this novel disease might have led to a low threshold towards inpatient admission. Younger data give a hospitalization rate of around 2.1%, which appears consistent with our proportion [52]. Furthermore, the SARS-CoV-2 vaccination rate in the general population and specifically in our cohort (91.7% at least once vaccinated) surely leads to relevant protection from severe COVID-19 courses. Additionally, the disease severity of COVID-19 decreased with the occurrence of new variants such as omicron [53, 54]. Other confounding factors (e.g., comorbidities besides IMID) for the course of COVID-19 were not considered in our statistical estimations—mainly due to very low absolute numbers (Table 12)—but at least demographic factors of our analysed patients are coherent when compared to population-based studies regarding IMID [55–58]. Fatal courses were obviously not detected as a deceased patient would not have been able to attend scheduled appointments and be screened for this study. However, none of our physician staff noticed or had been informed about a single IMID patient who had died in direct association with COVID-19.

Therapeutic steroid use in COVID-19 is a huge and extensively studied issue of the pandemic. Downregulation of a pathogenic *cytokine storm* was discussed as beneficial in certain subgroups during acute COVID-19 [59]. Nevertheless, chronic steroid use—which means *pre*-existing to SARS-CoV-2 infection—was associated with symptomatic disease and risk for a severe course of COVID-19. That could be shown in people with rheumatic diseases, in the IBD population and in nationwide studies [18, 23, 60]. An increased risk of symptomatic infection has been derived from these findings. However, data for a higher susceptibility to SARS-CoV-2 under prior steroid use are lacking. Due to the nature of COVID-19 with inconsistent testing strategies and high rates of asymptomatic infections, standard epidemiological methods hardly might ever close that gap of knowledge. On the other hand, steroid recipients—like in our cohort—might be more likely to adhere to isolation precautions, thereby reducing their overall exposure which leads to underestimation of their susceptibility to SARS-CoV-2. This could analogously in our case, lead to an association of protective systemic steroid therapy with regard to COVID-19.

Symptoms of COVID-19 and IMID condition might interfere: diarrhoea as a typical finding in IBD patients and muscle/joint pain in rheumatic patients are reported as a COVID-19 symptom in the general population, in IMID patients and in our study as well [24, 25, 61, 62]. Especially musculoskeletal symptoms are very common in post-COVID-19 patients too: approx. 30% of post-COVID-19 patients fulfil the criteria for fibromyalgia [63]. Answers in that particular context have to be taken very cautiously. The distinction between IMID disease flares and COVID-19 is, therefore, considered very challenging. Involved medical associations tried to face this issue with guideline recommendations [64–66]. Post hoc estimation of whether IMID activity and/or COVID-19 was contributing to the above-mentioned specific symptoms could not be addressed by our study approach and required a more objective methodology. Global quality of life had been assessed as a visual analogue scale: a history of COVID-19 did not seem to have an influence on that specific topic (data not shown).

These biases were a principal problem of this self-reported, questionnaire-based study: the long time since the course of COVID-19 might have limited memory of specific symptoms at that time. Further relevant limitation is given through the monocentric approach of our study, which might have led to a selection bias. Besides, as the study was conducted by our gastroenterologic/rheumatic ward, no further localization of the IMID spectrum, such as ophthalmologic or dermatologic involvement, is presented. This might limit generalisability, but nevertheless, our study approach gives a good overview of the situation in a significant patient collective. Additionally, our study is conceptualized prospectively which improves information quality in general, although individual data collection has to be seen in retrospect.

IMID patients represent a special population in the context of COVID-19. Our study presents an overview of a large-scale medical center during a pandemic situation to elucidate this topic.

## Figures and Tables

**Table 1:** Patient distribution of rheumatic and inflammatory bowel diseases.

Characteristic	No.	%
Total	156	100
Inflammatory bowel disease	117	75.0
Crohn's disease	69	44.2
Ulcerative colitis	48	30.8
Rheumatic disease	39	25.0
Rheumatoid arthritis	13	8.3
Axial spondyloarthritis	9	5.8
Systemic lupus erythematosus	6	3.8
Psoriatic arthritis	6	3.8
Granulomatosis with polyangiitis	2	1.3
Sjögren's syndrome	1	0.6
Juvenile idiopathic arthritis	1	0.6
Dermatomyositis/polymyositis	1	0.6

**Table 2:** Patient characteristics of the entire cohort.

Characteristic	No.	%
Total	156	100
Inflammatory bowel disease	117	75.0
Rheumatic disease	39	25.0
Age (years)		
Median	42	
Mean	46	
Range	18–95	
Male	67	42.9
Female	89	57.0
Unvaccinated against SARS-CoV-2^§^	10	6.4
Vaccinated against SARS-CoV-2^§^		
One vaccination	143	91.2
Two vaccinations	136	87.2
Three vaccinations	113	72.4
Four vaccinations	11	7.1
Self-reported history of COVID-19^†^		
No	72	46.2
Yes	81	51.9

^§^Data obtained by 153 patients; SARS-CoV-2: severe acute respiratory syndrome coronavirus 2; COVID-19: coronavirus disease 2019; ^†^data obtained by 153 patients.

**Table 3:** Administered vaccines against SARS-CoV-2.

	No.	%
Entire cohort	156	100
Unvaccinated	10	6.4
Unknown vaccination status	3	1.9
Vaccinated/number of vaccinations		
First vaccine	143	91.7
Vaxzevria® (AstraZeneca AB)	12	
Comirnaty® (BioNTech GmbH)	114	
Jcovden® (Johnson & Johnson)	4	
Spikevax® (Moderna Biotech)	13	
Second vaccine	136	87.1
Vaxzevria®	4	
Comirnaty®	113	
Spikevax®	18	
Unknown	1	
Third vaccine	113	72.4
Vaxzevria®	3	
Comirnaty®	83	
Spikevax®	25	
Unknown	2	
Fourth vaccine	11	7.1
Comirnaty®	8	
Spikevax®	2	
Unknown®	1	

SARS-CoV-2: severe acute respiratory syndrome coronavirus 2.

**Table 4:** Proof of SARS-CoV-2 infection.

	No	%
Total	81	100
Polymerase chain reaction (PCR)	68	83.9
Antigen test only	5	6.2
Antibody test only	1	1.2
Other (e.g., clinical suspicion)	4	4.9
No proof provided	3	3.7

The percentage refers to patients with self-reported COVID-19; SARS-CoV-2: severe acute respiratory syndrome coronavirus 2.

**Table 5:** Severity of COVID-19.

	No.	%
Total	81	100
No special treatment	64	79.0
Outpatient (e.g., general practitioner)	15	18.5
Inpatient ward	1	1.2
Intensive care unit	1	1.2

The percentage refers to patients with self-reported COVID-19; SARS-CoV-2: severe acute respiratory syndrome coronavirus 2.

**Table 6:** Patient characteristics.

	With history of COVID-19^‡^		Without history of COVID-19^‡^		Chi^2^	*t* test^†^
	No.	%	No.	%		
	81	100	72	100		
Age (years)						
Median	39.0		47.0			0.05^*∗*^
Mean	42.9		48.2			
Range	18–84		18–82			
Male	33	40.7	32	44.4	0.77	
Female	48	59.3	40	55.6		
Rheumatic disease	24	29.7	14	19.4	0.20	
Inflammatory bowel disease	57	70.3	58	80.6		
Unvaccinated against SARS-CoV-2^§^	8	9.8	2	2.7	0.14	
Vaccinated against SARS-CoV-2^§^						
One vaccination	71	87.7	69	95.8	0.46	
Two vaccinations	65	80.2	68	94.4		
Three vaccinations	47	58.0	64	88.9		
Four vaccinations	3	3.7	6	8.3		

3 patients did not provide information about their history of COVID-19; ^†^welch modification; ^‡^data obtained by 153 patients; ^§^data obtained by 150 patients; COVID-19: coronavirus disease 2019; SARS-CoV-2: severe acute respiratory syndrome coronavirus 2; ^*∗*^*p* < 0.05.

**Table 7:** Symptoms of COVID-19.

	No.	%
Self-reported COVID-19	81	100
No symptoms	7	8.6
Fatigue/tiredness	58	71.6
Common cold	55	67.9
Cough	51	62.9
Headache	44	54.3
Fever	35	43.2
Sore throat	33	40.7
Joint pain	31	38.2
Muscle pain	28	34.6
Taste disorder	21	25.9
Diarrhoea	20	24.6
Anosmia	19	23.4
Vertigo	18	22.2
Dyspnea	15	18.5
Memory disorder	13	16.0
Dry mucosa	12	14.8
Chest pain	11	13.6
Nausea	10	12.3
Painful breathing	4	4.9
Hypersalivation	3	3.7

Other: chills (2x), paresthesia (1x), cognitive dysfunction (1x), and gastrointestinal bleeding (1x). COVID-19: coronavirus disease 2019; multiple answers were possible.

**Table 8:** Symptoms of COVID-19 regarding rheumatic and inflammatory bowel diseases.

	Entire cohort		Rheumatic disease		Inflammatory bowel disease	
	No.	%	No.	%	No.	%
COVID-19	81	100	24	100	57	100
No symptoms	7	8.6	1	4.2	6	10.5
Fatigue/tiredness	58	71.6	19	79.2	39	68.4
Common cold	55	67.9	17	70.8	38	66.7
Cough	51	63.0	18	75.0	33	57.9
Headache	44	54.3	14	58.3	30	52.6
Fever	35	43.2	9	37.5	26	45.6
Sore throat	33	40.7	12	50.0	21	36.8
Joint pain	31	38.3	8	33.3	23	40.4
Muscle pain	28	34.6	9	37.5	19	33.3
Diarrhoea	20	24.7	5	20.8	15	26.3
Anosmia	19	23.5	4	16.7	15	26.3
Taste disorder	21	25.9	10	41.7	11	19.3
Vertigo	18	22.2	4	16.7	14	24.6
Dyspnea	15	18.5	7	29.2	8	14.0
Memory disorder	13	16.0	4	16.7	9	15.8
Dry mucosa	12	14.8	6	25.0	6	10.5
Chest pain	11	13.6	3	12.5	8	14.0
Nausea	10	12.3	4	16.7	6	10.5
Painful breathing	4	4.9	1	4.2	3	5.3
Hypersalivation	3	3.7	1	4.2	2	3.5

COVID-19: coronavirus disease 2019; multiple answers were possible.

**Table 9:** Analysis of IMID patients regarding COVID-19 free survival.

	No.	%	Observed COVID-19 cases	Expected COVID-19 cases	Logrank test
Total	156	100			
Unvaccinated	10	6.4	8	4.1	Chi^2^ = 4.1
Vaccinated^§^	137	87.8	68	71.9	*p*=0.04^*∗*^

IMID: immune-mediated inflammatory disease; COVID-19: coronavirus disease 2019; ^§^at least one vaccination against severe acute respiratory syndrome coronavirus 2 (SARS-CoV-2); 3 patients did not provide vaccination status, and 6 patients did not provide sufficient data to calculate event time; ^*∗*^*p* < 0.05.

**Table 10:** Systemic pharmacotherapy for the immune-mediated inflammatory disease at the time of survey.

	With history of COVID-19^†^			Without history of COVID-19^†^			Chi^2^
	No.	(IBD/RD)	%	No.	(IBD/RD)	%	
	81		100	72		100	
Azathioprine	12	(12/0)	14.8	11	(9/2)	15.3	1
Mycophenolate	5	(0/5)	6.2	0		—	0.09
Methotrexate	5	(0/5)	6.2	5	(0/5)	6.9	1
Hydroxychloroquine	3	(0/3)	3.7	1	(0/1)	1.4	0.70
Systemic glucocorticoid	11	(3/8)	13.6	22	(13/9)	30.6	0.02^*∗*^
Biologic agent	35	(23/12)	43.2	36	(30/6)	50.0	0.50
Adalimumab	9	(6/3)	11.1	14	(13/1)	19.4	
Infliximab	11	(8/3)	13.6	6	(4/2)	8.3	
Etanercept	2	(0/2)	2.5	0		—	
Ustekinumab	2	(1/1)	2.5	2	(2/0)	2.8	
Vedolizumab	8	(8/0)	9.9	11	(11/0)	15.3	
Rituximab	1	(0/1)	1.2	2	(0/2)	2.8	
Belimumab	2	(0/2)	2.5	0		—	
Abatacept	0		—	1	(0/1)	1.4	
Janus kinase inhibitor (e.g., upadacitinib)	2	(0/2)	2.5	2	(0/2)	2.8	1

^†^data obtained by 153 patients; COVID-19: coronavirus disease 2019; IBD: inflammatory bowel disease; RD: rheumatic disease ^*∗*^*p* < 0.05.

**Table 11:** Systemic glucocorticoid therapy of IMID patients at the time of survey.

	With history of COVID-19^†^		Without history of COVID-19^†^		Chi^2^	*t* test
	No.	%	No.	%		
	81	100	72	100		
Systemic glucocorticoid^‡^	11	13.6	22	30.6	0.02^*∗*^	
Dosage (mg)						
Mean	10.5		10.3			0.98
Median	5		5			
Range	1–60		2–50			

IMID: immune-mediated inflammatory disease; COVID-19: coronavirus disease 2019; ^*∗*^*p* < 0.05; ^†^data obtained by 153 patients; ^‡^to facilitate calculations, all individual dosages were standardized by conversion to a prednisone equivalent [28].

**Table 12:** Relevant comorbidities for severe COVID-19.

	No.	%
Total	156	100
Hypertension	13	8.3
Lung disease (e.g., COPD and asthma)	5	3.2
Diabetes	4	2.6
Coronary heart disease	4	2.6
Cancer (active disease/treatment)	1	0.6
Dilated cardiomyopathy	1	0.6

COVID-19: coronavirus disease 2019; COPD: chronic obstructive pulmonary disease.

## Data Availability

The data used to support the findings of this study are included within the article.
